# How many days are needed for a reliable assessment by the Sleep Diary?

**DOI:** 10.5935/1984-0063.20190131

**Published:** 2020

**Authors:** Diego de Alcantara Borba, Raquel Sousa Reis, Pedro Henrique Tadeu de Melo Lima, Lucas Alves Facundo, Fernanda Veruska Narciso, Andressa Silva, Marco Túlio de Mello

**Affiliations:** 1 Universidade do Estado de Minas Gerais, 1Departamento de Ciências do Movimento Humano - Ibirité - Minas Gerais - Brazil; 2 Universidade Federal de Minas Gerais, Departamento de Esportes, Escola de Educação Física, Fisioterapia e Terapia Ocupacional - Belo Horizonte - Minas Gerais - Brazil

**Keywords:** Sleep, Sleep Deprivation, Surveys, Questionnaires

## Abstract

**Introduction:**

Population sleep registration is important for the epidemiological investigation of possible disorders and diseases associated with sleep deprivation and restriction. The aim of the current study was to verify how many days and weeks are required for a reliable sleep diary application.

**Methods:**

53 subjects of both genders participated in the study (Age: 25±9 years). The volunteers answered the morning-evening questionnaire for later identification of their chronotype (21 mornings; 22 undetermined; 10 evenings). They then received the sleep diary to fill in for a period of 28 consecutive days (or 4 weeks) of their nighttime sleep. The participant was instructed to describe the time at which they started sleeping and woke up within one hour after waking up.

**Results:**

There was no difference in participants’ sleep time between weeks. The participants had longer sleep times on Sunday and Saturday compared to other days of the week. The sleep diary reliability values increased with the increasing number of nights, reaching adequate reliability (≥ 0.70) with seven consecutive days recorded.

**Conclusion:**

A sleep diary seems to be a reliable tool for assessing adult sleep time, especially when used for a period of at least seven consecutive days including weekends.

## INTRODUCTION

Recording the population’s sleep is important investigating disorders and diseases associated with sleep loss^1^. A significant number of people currently have a decreased sleep duration and quality. This situation can lead to the onset of disorders such as daytime sleepiness, increased stress and irritability^1^.

The most frequently implemented instruments for sleep analysis are polysomnography (recording of brain electrical activity by electrodes), which is considered the gold standard, and actigraphy (recording the sleep-wake cycle by means of a wrist accelerometer, where low activity indicates the sleep period). However, these methods are usually only performed in specialized clinics or hospitals, they require a specialized professional and have a relatively high cost. From a population point of view, these methods considerably limit the number of people assessed.

An alternative to sleep study is the application of specific questionnaires about sleep quality and using a sleep diary^2,3^. A sleep diary (SD) consists of daily recording of information by the participant about their sleep habits, wakefulness and general activities during a certain period of nights/days^2^. According to some authors, subjective sleep recording may be less accurate than more objective assessments (i.e. polysomnography and actigraphy)^2,3^. However, these assessment instruments using questionnaires are considered low cost, simple to apply and reliable for evaluating sleep and wake periods^1,2^. Rogers et al.^4^ found a high concordance rate of sleep time recorded through a sleep diary and polysomnography (kappa = 0.87), as well as high sensitivity and specificity (92.3% and 95.6%, respectively). In addition, subjective evaluations have lower cost advantages and are easier to apply in field research, clinical evaluations and studies applied with large and small numbers of participants^5^.

Although SD is widely used in the scientific field for monitoring sleep and wake habits, there is no consensus among researchers about the amount of recording time needed to safely and reliably assess sleep habits^2^. Some authors have used the SD for short periods of less than one week^6^, while others have used it for seven or more days^7,8^. For example, in a study with children and adolescents, Short et al.^9^ showed that the adequate recording night number depends on the resident country (mean of five nights were required to achieve adequate reliability). However, the number of nights to reach this value varied by residence country and only evaluating the weekend (Saturday and Sunday) presented inadequate reliability. It is important to emphasize that Short et al.^9^ did not cover South American countries.

Inaccurate application of the sleep diary in terms of quantity may result in misinterpreting the results and compromise analysis. Thus, the effect of recording the number of days as well as the specific weekdays required for a reliable analysis of sleep habits is controversial and should be further clarified. Therefore, the aim of the present study was to check how many days and weeks are necessary for a reliable SD application. The SD is an important and widely-used instrument by researchers and clinicians interested in understanding the quantity and quality of sleep. Therefore, better understanding on how to apply this instrument is fundamental for better interpretation and diagnosis of its results.

## METHODS

Ethical approval for the study was granted by the Federal University of Minas Gerais Research Ethics Committee. The participants included 53 college students (25±9 years) of both genders recruited from two Brazilian universities located in the state of Minas Gerais, Brazil. All students were eligible to participate and there were no exclusion criteria. Informed consent was obtained from the participants.

### Procedures

The researcher invited the participants. The participants who agreed to participate answered the morningness-eveningness questionnaire for later identification of their chronotype^9^. This questionnaire is a subjective instrument composed of questions regarding the respondent’s preferential period (morning, afternoon or evening) to perform daily activities, such as working, studying, exercising, sleeping and waking up. Thus, individuals can be classified by the following scores: morning individuals (score > 58); intermediate or indifferent (score 42 to 58) and afternoon (score < 42)^10,11^.

Then the participant received the SD to complete for a period of 28 consecutive days (4 consecutive weeks). Participants completed a pencil and paper sleep diary about their night sleeping and waking-up schedules. The SD was structured and its application followed the norms suggested by Carney et al.^2^, in which the participant should record their time of falling asleep and waking up in the sleep diary within one hour after awakening. Week sleep time was determined by the average daily sleep time of participants. The analysis and comparison of sleep time between days of the week were evaluated by the average value of four records of the week specific days (e.g. average of four Monday data), since the sleep diary was applied over a four-week period. The difference between bed time and sleep time was explained to the participants in order to minimize the sleep diary record mistakes. The participant was instructed to record their sleep time and not their time bed.

### Statistical analysis

Data were expressed as mean ± standard deviation and confidence interval (95% CI). Data normality was analyzed using the Shapiro-Wilk test. One-way repeated measures ANOVA was used to compare total sleep time between weeks and days. Tukey’s post hoc was used to identify if any differences were found. The significance level adopted was less than 5%. The night effect number on the sleep time reliability was determined by calculating the intra-class correlation coefficient for one-way repeated measures ANOVA. These reliability coefficients indicate the proportion of true measure variance in the aggregate results each night, with coefficients above 0.70 indicating adequate reliability^12^. Analyzes were performed using the SPSS 20.0 program.

## RESULTS

**Figure 1:**
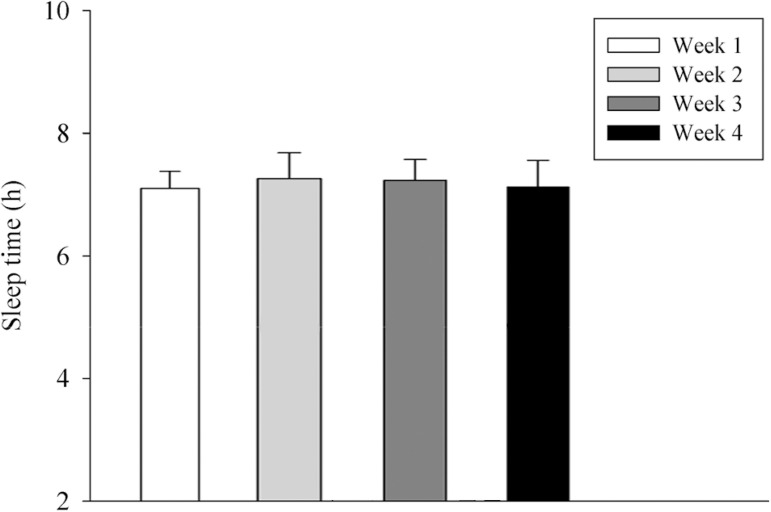
Mean ± sd of sleep time among the weeks. ANOVA one way. F= 1.04; p= 0.40.

Twenty-one participants were classified as morning, 22 undetermined and 10 evening chronotypes. There was no difference in the participants’ sleep time among weeks. [week 1: 7.10±0.30 (CI_95%_= 7.02-7.18); week 2: 7.30±0.40 (CI_95%_= 7.19-7.41); week 3: 7.20±0.30 (CI_95%_= 7.12-7.28); week 4: 7.10±0.40 (CI_95%_=6.99-7.21); F=1.04; *p*=0.40] (Figure 1).

**Figure 2:**
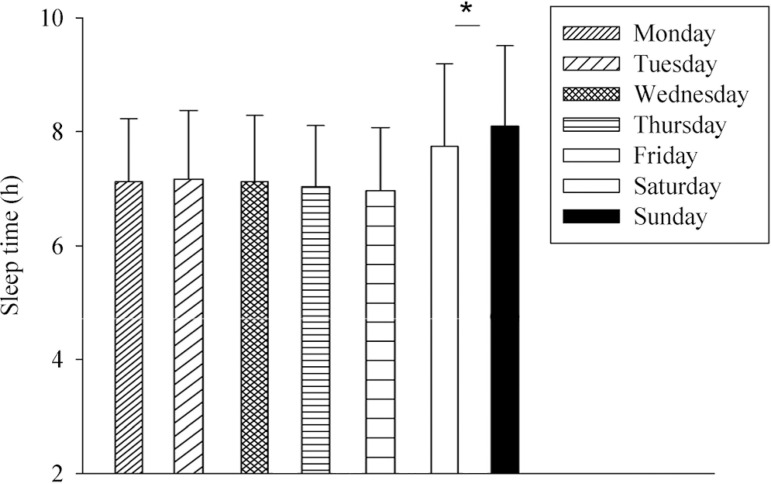
Mean ± sd of sleep time among the week days. ANOVA one way. F= 6.038; p< 0.001. (*) higher than Monday, Tuesday, Wendnesday Thursday and Friday.

The participants presented higher sleep time for the Saturday and Sunday (8.10±1.40h, CI_95%_=7.72-8.48; 7.70±1.40h, CI_95%_=7.32-8.08, respectively) compared to Monday (7.10±1.10 h, CI_95%_=6.8-7.4), Tuesday (7.20±1.20 h, CI_95%_=6.87-7.52), Wednesday (7.10±1.10 h, CI_95%_=6.8-7.4), Thursday (7.01±1.01 h, CI_95%_=6.73-7.27) and Friday (7.01±1.11 h, CI_95%_=6.70-7.30). There was no difference between Saturday and Sunday sleep time (*p*>0.05) (Figure 2).

Table 1 shows the effect size value among the days of the week. The results suggested moderate and large effect size for the weekend compared to weekdays.

**Table 1:** Effect size according to the week days.

Week days	Tuesday	Wednesday	Thursday	Friday	Saturday	Sunday
Monday	0.08	0.0	0.10	0,10	0.48	0.80
Tuesday	-	0.08	0.18	0.17	0.38	0.70
Wednesday	-	-	0.10	0.10	0.48	0.80
Thursday	-	-	-	0.0	0.58	0.92
Friday	-	-	-	-	0.56	0.88
Saturday	-	-	-	-	-	0.28

The estimated reliability for the aggregate values according to the successive number of recorded nights (1 to 28) is shown in Table 2. As shown, the greater the number of aggregate nights, the greater the reliability (i.e. seven night values are more reliable than the values of four nights). Adequate reliability (≥ 0.70) was achieved on the seventh night.

**Table 2:** Reliability of measures aggregated number of nights.

Number of nights and its respective coefficient value						
**1**	**2**	**3**	**4**	**5**	**6**	**7**
-	.44	.62	.55	.60	.67	.70
**8**	**9**	**10**	**11**	**12**	**13**	**14**
.76	.77	.77	.78	.78	.80	.81
**15**	**16**	**17**	**18**	**18**	**20**	**21**
.83	.84	.85	.85	.87	.87	.88
**22**	**23**	**24**	**25**	**26**	**27**	**28**
.88	.89	.90	,91	.91	.91	.92

*Above (bold)=nights number. Below (*italic*)=respective coefficient value.

## DISCUSSION

The present study investigated the total time required for SD application. In addition, it compared the sleep time among the weeks and days of the week in young adults. The SD is widely used to assess sleep and wakefulness habits and daily activities in general, as it has low cost, easy access and application^1,4^. However, aspects related to the reliable application time need to be further elucidated.

Guidelines on the time of SD application are presented in the literature through consensus and norms^2,3^. However, studies in the literature which in fact infer about the effectiveness of the SD application on different days and weeks are scarce. Our findings show that sleep time was similar among consecutive weeks. These results suggested one week (7 days) of SD application is sufficient for reliable assessment of sleep time. This appears to be the first study comparing adult sleep time between four consecutive weeks of SD application.

On the other hand, the specific day of the week seems to influence the sleep time, in which the weekends (Saturday and Sunday) presented higher sleep time values​ than the weekdays. The participants of this study were students with night classes with their classes ending around 10:15 pm, and also students with daytime classes, with classes beginning at 07:30 am. In addition, most students (90%) with night classes worked during the day, which may explain their shorter sleep time on school/work days compared to weekend days (Sunday) when sleeping and waking hours are less compromised.

These results corroborate those of Wolfson et al.^13^, who found a longer weekend sleeping time compared to weekdays in 302 students (mean = 16 years) of both genders, as well as those of Short et al.^9^ who found longer sleep on the weekend in a study with 1766 young people (10-18 years) from different countries. the SD was applied for seven consecutive days in both studies. These authors, as well as those of the present study, attribute this longer sleep time on weekends to greater flexibility in bedtime and waking up, and especially because they did not have classes on those days. Therefore, it seems more consistent that the SD application also includes Saturdays and Sundays for a safer and more accurate interpretation of the sleep habits of the investigated population.

Also in this context, the present study also evaluated the consecutive night reliability of SD application. The appropriate reproducibility reference value of 0.7 was reached on the seventh evaluation night. This indicates that seven days of SD are sufficient for safely determining young adults’ sleep time. To our knowledge, only one study evaluated the SD consistency on sleep time over consecutive days. In a study with young people (10-18 years) from different countries, Short et al.^9^ found five nights overall were required to achieve adequate reliability (0.7).

However, the number of nights to reach this value varied by residence country. For example, young Americans achieved a 0.7 reliability value with three consecutive weekday nights. An evaluation of only the weekend (Saturday and Sunday) presented inadequate reliability (0.08 - 0.43), and the value range varied according to the participants’ nationality. In addition, the study by Short et al.^9^ did not cover South American countries, increasing the relevance of the present study. Thus, seven subsequent nights of recording in a sleep diary related in the present study as in Short et al.^9^ were sufficient to provide an adequate and reliable evaluation of sleep time.

A previous study using the actigraphy technique was performed to evaluate the reliability of consecutive days on some sleep variables and indicated similar results about how many days are required for a reliable evaluation of sleep time. Acebo et al.^14^ showed that reliability estimates for mean aggregate values of seven recorded nights were adequate for total sleep time in adolescents of both genders. Proper reliability is important for clinical applications, as poor data reliability can result in misdiagnosis. Therefore, the most reliable answers about the sleep habits of a particular patient or group of people should have at least seven nights of evaluation.

Although the literature indicates few studies which in fact measured the effects of the nights number on adequate sleep evaluation, most studies use approximately seven evaluation nights^7,14,15^, without appropriate justification. However, those who use less than seven assessment nights to characterize sleep time or other wake/sleep cycle variables are less frequent^6^. As seen, a seven-day period of SD is adequate to infer night sleep time, but unlike the studies mentioned above, the present study made it possible to understand that the addition of more days of sleep diary application is able to modify the reliability values ​​for this measurement. As expected, the addition of more days/nights of analysis were accompanied by an increase in reliability values ​​from 0.70 on the seventh day to 0.81 on the 14^th^, 0.88 on the 21^st^, and 0.92 on the 28^th^ day. However, it is entirely possible to record the SD for only 7 days (reliability = 0.70), if patients and/or research participants have less time to perform such recording.

The present study provides important information to researchers, clinicians, and other groups of professionals interested in assessing sleep through the SD. In addition, this was the first study to evaluate the reliability of using this instrument for a relatively longer period (one month), further reinforcing the SD as a reliable tool for estimating sleep time. On the other hand, our findings cannot be extrapolated to other populations such as children, older adults, people with sleep disorders or associated diseases. In addition, the results of the present study lack information on the validity of the SD for other important variables such as latency, wakefulness after sleep and sleep efficiency, as these variables are fundamental for classifying sleep quality.

## CONCLUSION

According to the findings of the present study, the sleep diary is a reliable, low-cost and far-reaching tool to assess the sleep time in the Brazilian population. The sleep diary should be applied for a period of at least seven days, including a weekend in order to enable a better sleep time assessment, as sleep time was similar between four consecutive weeks. However, Saturday and Sunday should be included in the sleep diary days for application, as the sleep time on weekends was different from the other days.
